# TRAINING LONG-TERM SURVIVORS OF HIV TO ENGAGE IN RESEARCH TO IDENTIFY PRIORITIES FOR AGING WITH HIV

**DOI:** 10.1093/geroni/igac059.1279

**Published:** 2022-12-20

**Authors:** Andy Rapoport, Rachel Lessem, Margaret Danilovich

**Affiliations:** Leonard Schanfield Research Institute, CJE SeniorLife, Chicago, Illinois, United States; CJE SeniorLife, Chicago, Illinois, United States; Leonard Schanfield Research Institute, Chicago, Illinois, United States

## Abstract

With effective antiretroviral therapies, HIV has become a chronic disease. Currently, >50% of people living with HIV are 50+ years old, and they face dual challenges of aging and HIV management. Overall, <15% of published studies in HIV engage stakeholders in research. These rates are even lower for older adults with HIV. Thus, there is a critical need to engage long-term survivors in developing meaningful research questions for aging with HIV. To address this gap, we created the SHARE (Survivors of HIV Advocating for Research Engagement) board. Presenters will discuss the process for building the board’s research capacity and results of a community needs assessment that board members designed and conducted to ascertain priorities for HIV-aging research. Presenters will discuss evidence-based educational strategies utilized to build member research knowledge, and pre-post training changes in knowledge, confidence, and understanding. Presenters will focus on novel training approaches implemented in the remote environment.

